# Mortality disparities among children and adolescents with tuberculosis in Tanzania Mainland from January 2023 to December 2023: A retrospective cohort study

**DOI:** 10.1371/journal.pgph.0005184

**Published:** 2025-10-30

**Authors:** Peter Richard Torokaa, Agricola Joachim, Daudi E. Komba, James N. Allan, Thobias Bolen, Onduru G. Onduru, Robert Balama, Riziki M. Kisonga, Allan N. Tarimo, Joakim Chacha, Mtebe Majigo

**Affiliations:** 1 President’s Office, Regional Administration and Local Government, Dodoma, Tanzania; 2 Tanzania Field Epidemiology and Laboratory Training Program, Dar es Salaam, Tanzania; 3 Muhimbili University of Health and Allied Sciences, Dar es Salaam, Tanzania; 4 Bunda District Hospital, Mara, Tanzania; 5 Tabora District Hospital, Tabora, Tanzania; 6 Ministry of Health, National TB and Leprosy Programme, Dodoma, Tanzania; 7 Ministry of Health, Diagnostic and Healthcare Technical Services Unit, Dodoma, Tanzania; Universidad de Chile, CHILE

## Abstract

Every year, over 10 million people worldwide contract tuberculosis (TB). The 2024 World Health Organisation TB global report indicated that 32% of the total deaths were children and adolescents under 15 years old. The scale of TB highlights the urgent need for action to end the global epidemic by 2030. This study aims to evaluate the mortality rate, survival probabilities, and factors associated with mortality among children and adolescents with TB in Tanzania. A retrospective cohort study was conducted from the Tanzania National Tuberculosis and Leprosy Programme data, which included individuals under 15 years old who began TB treatment between 1^st^ January 2023 and 31^st^ December 2023. The last patient’s end-of-follow-up time was on 16^th^ June 2024. The primary outcome of interest in our study was death. We calculated overall and covariate-specific TB mortality rates per 1,000 person-months. The Kaplan-Meier curve was employed to estimate survival probabilities. A total of 10,491 children and adolescents receiving TB treatment were included, nearly half of whom, 5,940 (56.62%), were under age 5 years. A total of 177 (1.69%) died, resulting in a crude mortality rate of 2.86 per 1,000 person-months. Furthermore, TB and HIV co-infection individuals had five times the risk of death (aHR = 5.03, 95% CI = 3.40-7.47, p < 0.001) compared to non-HIV infection. Community referrals were associated with a lower risk of mortality (aHR = 0.54, 95% CI = 0.35-0.84, p = 0.006). We observed significantly lower survival probabilities for patients referred from CTC, with rates of 96.8%, 96.0% and 95.8% at 2, 4, and 6 months, respectively, compared to those referred from the community, which showed higher survival probabilities of 99.5%, 99.3% and 99.2% over the same periods. The findings reveal significant differences in TB mortality in relation to age, referral system and co-infection. Integrating TB services with child healthcare programs and strengthening differentiated service delivery models can improve survival rates. Targeted interventions in high-risk areas are essential to reduce TB mortality.

## Introduction

Tuberculosis (TB) is a global public health issue that affects millions of people and necessitates international collaborative efforts for its elimination [1]. According to the WHO Global Tuberculosis Report 2024, 39 countries showed an increase in TB incidence rates in 2023 that was more than 5% higher than in 2015, with Tanzania among the three countries with high burden TB projected to be nearing the achievement of the 2025 milestone [2]. Children and adolescents under 15 years old represent approximately 11% of global TB cases, with nearly half being under five years old [3]. From 2015 to 2022, the global decline in TB cases was only 8.7%, significantly below the 50% target set by the World Health Organization (WHO) to be achieved by the end of 2025 [4]. An estimated 214,000 children and adolescents under 15 years old died from TB in 2022 [5].

Globally, there is limited published data on the burden and outcomes of pediatric TB [6]. Most available reports on TB outcomes relate more to adults than to children [7]. This variation may be attributed to the difficulties in diagnosing pediatric TB, especially in developing countries [6,8]. However, some studies in Africa have consistently reported high mortality rates among children and adolescents with tuberculosis (TB). For instance, a study conducted in Ethiopia found that pediatric TB patients had a significantly higher risk of mortality compared to adults [9]. A study in South Africa has highlighted the impact of TB on children and adolescents, emphasizing the need for improved diagnostic and treatment strategies [10]. The accuracy of measuring TB-related mortality in developing countries is challenging due to the lack of vital registration systems in many countries [11]. Consequently, mortality estimates in these countries are derived indirectly by multiplying the estimated TB incidence by the case fatality rates recorded in established cohorts of patients enrolled in treatment programs [12,13]. The factors associated with TB mortality among children and adolescents vary across countries. Several studies in African countries reported that human immunodeficiency virus (HIV), in children under five years, has a severe clinical state upon admission and extrapulmonary TB is reported as the main factor for TB mortality [14–17].

The 2015–2023 National Tuberculosis and Leprosy Programme (NTLP) TB notification report in Tanzania indicated that pediatric cases represented approximately 7.4% to 15.8% respectively of all reported TB cases [18]. Around the same period, it was reported that nearly 200,000 children under 5 years old died from Tuberculosis (TB) in sub-Saharan Africa and Southeast Asia [19]. In response to the burden of TB faced by children, NTLP developed the guideline for managing pediatric TB to tackle the challenges of treatment and care. The treatment regimen in the new 7^th^ edition guideline of April 2020 includes a two-month intensive phase for first line drugs which includes the isoniazid, rifampicin, pyrazinamide and ethambutol followed by a continuation phase lasting at least four months with isoniazid and rifampicin, however, the doses of first-line anti-TB agents differs from those administered in adults. Child-friendly, fruit-flavored TB medications that are dispersible in liquid and tasty significantly improve the success of administering TB treatments to children. Furthermore, the guidelines emphasize the importance of prompt initiation of therapy and adequate nutrition to enhance the immune response and achieve favorable treatment outcomes [20].

We conducted this study to summarize the status of pediatric TB in Tanzania in terms of mortality rates, survival probabilities, and factors associated with pediatric TB mortality. The information presented in this study underscores the progress made in addressing the burden of pediatric TB in Tanzania and the factors that should be considered for effective interventions.

## Materials and methods

### Ethics statement

The Senate Research and Publications Committee of Muhimbili University of Health and Allied Sciences granted ethical permission and waived the informed consent (Ref. No. DA.282/298/02L/499). Additionally, the Ministry of Health’s (MoH) Permanent Secretary approved access to data at the national level. Data were routinely collected by health facilities that provide TB care and treatment services, with strong emphasis on ethical standards including patients consent. All data were fully anonymized before they are accessed.

### Study design, setting and population

We conducted a retrospective cohort study using data from the Tanzania NTLP database, which routinely collects information from TB and Leprosy treatment centers across all 26 regions of mainland Tanzania. The study included children and adolescents under 15 years who began TB treatment between 1^st^ January and 31^st^ December 2023, excluding those with missing treatment outcome information or inconsistent treatment dates. The discrepancies or errors in the recorded start and end dates of TB treatment that made it impossible to accurately assess treatment duration or outcomes were considered inconsistent treatment dates. These include: missing start or end dates, illogical sequences (e.g., treatment end date before start date), overlapping or duplicated treatment records.

### TB referral and treatment system

Tanzania has a well-organized TB referral and treatment system designed to ensure widespread access and effective management of TB cases. Individuals may enter the TB care through various channels, including self-referral, in which patients seek care on their own at health facilities; community referrals, in which patients are referred by community health workers or volunteers to TB services; HIV care clinics (CTC), patients are referred from HIV Care and Treatment Clinics (CTC), often due to TB symptoms identified during HIV care; and other sources such as traditional healers or pharmacies. Diagnosis and treatment begin at designated TB diagnostic centers, where confirmed cases are registered for monitoring. Treatment is guided by national protocols aligned with WHO standards, and all patients are required to undergo Directly Observed Therapy (DOT). Facility-based DOT is recommended for patients with severe illness or poor adherence, where health workers supervise daily medication intake. For stable patients, home-based DOT is an option, involving a trusted treatment supporter who oversees drug administration. These patients must visit the health facility weekly during the intensive phase and biweekly during the continuation phase for follow-up and medication refills [20].

### TB and leprosy data management system

Tanzania use the District Health Information Software 2 (DHIS2), which includes the module of Electronic TB and Leprosy (eTL) register for TB notifications. All data are entered per each TB case notified. Data for drug-resistant and drug-susceptible TB is entered in the eTB register for TB reporting. Patient-level data, including information of the individual TB patient such as demographics, diagnosis, treatment progress, and outcomes, are documented and reported quarterly. The purpose of these data is for monitoring treatment outcomes, evaluating program performance, resource planning, and Policy and strategy development. For high-volume facilities, this data is also reported daily in the register from the health facility to the national level.

The TB notification process is a structured part of the national surveillance system. Health care workers at designated TB diagnostic and treatment centers are responsible for recording TB cases. This includes clinicians, nurses, and TB focal persons trained in TB case management and reporting. Data are recorded once a patient is confirmed to have TB and their details are entered into the TB register. The follow-up data, including treatment progress and outcomes, are recorded regularly. Reports are submitted monthly to district, regional and national levels for aggregation and analysis.

### Data collection

We extracted demographic and clinical information from the electronic TB and Leprosy register (eTL) database and then exported it to Microsoft Excel on 1^st^ March 2025. The independent variables were demographic characteristics (age, sex, residence, and health facility level) and clinical characteristics (TB referral type, TB treatment history, disease classification, directly observed therapy (DOT) options, and HIV status). The dependent variable was mortality during the treatment period.

### Data analysis

Data was analysed using Stata version 15.1 software. We analyzed categorical variables and presented them using frequencies and proportions, and reported the median (interquartile range, IQR) for continuous variables. Pearson’s chi-square test was used to compare categorical variables. We calculated overall and covariate-specific TB mortality rates per 1000 person-months and estimated survival probabilities using the Kaplan-Meier estimator and curve, respectively. The log-rank test was used to assess significant differences between the survival curves. Factors with a p-value ≤ 0.2 in the bivariate analysis were considered as potential risk factors and included in the multivariable analysis using the Cox proportional hazards model. The Cox proportional hazards model was applied to conduct univariate and multivariate analyses, with Schoenfeld’s test used to evaluate the proportional hazards assumption. Hazard ratios and their respective 95% confidence intervals were reported.

The primary outcome was death during TB treatment regardless of the cause of death, and the secondary outcome was survival probabilities at different time points (2, 4, and 6 months). We defined survival time as the period from the initiation of TB treatment until death or censoring (measured in months). Children and adolescents with TB were defined as those who began TB treatment between 1^st^ January and 31^st^ December 2023, along with the last follow-up of their TB treatment. The last patient’s end-of-follow-up time was on 16^th^ June 2024. The Children and adolescents were followed up from the date of TB treatment initiation until the completion of their TB treatment of 168 days or death. Individuals who were lost to follow-up or whose treatment was classified as a failure or as cured were censored based on the last follow-up date. Those who were alive at the completion of their TB treatment or the last follow-up date were also considered censored.

## Results

### Participant characteristics

A total of 10,491 children and adolescents were included in the study for analysis (S1 File). Most participants, 5,940 out of 10,491 (56.62%), were under the age of 5 years, and the majority, 5,476 (52.20%), were male. Among the participants, 6,008 (57.27%) lived in rural areas, with 7,765 (74.02%) having pulmonary TB and 4,273 (40.73%) being community referrals. Almost one third of participants, 3,767 (35.90%), attended hospitals, and 10,433 (99.45%) were new TB patients. The majority of participants, 10,384 out of 10,491 (98.98%), received the home-based DOT option, and 9,738 out of 10,491 (92.82%) were HIV negative (Table 1).

**Table 1:** Social-demographic and clinical characteristics of the study participants (n = 10,491).

Variables	Characteristics	Frequency n (%)
**Age (Years)**	<5	5,940 (56.62)
	5-9	2,419 (23.06)
	10-14	2,132 (20.32)
	*Median (IQR) years*	*4.0 (2 –8 )*
**Sex**	Male	5,476 (52.20)
	Female	5,015 (47.80)
**Residence**	Urban	4,483 (42.73)
	Rural	6,008 (57.27)
**Health facility level**	Dispensaries	3,637 (34.67)
	Health centres	3,087 (29.43)
	Hospitals	3,767 (35.90)
**TB referral type**	Self-referrals	3,746 (35.71)
	CTC referrals	404 (3.85)
	Community referrals	4,273 (40.73)
	Other referral types	2,068 (19.71)
**Treatment History**	New	10,433 (99.45)
	Retreatment	58 (0.55)
**Disease classification**	Pulmonary	7,765 (74.02)
	Extra pulmonary	2,665 (25.40)
	Both Pulmonary and extra pulmonary	61 (0.58)
**DOT option**	Home based	10,384 (98.98)
	Facility based	107 (1.02)
**HIV status**	Positive	753 (7.18)
	Negative	9,738 (92.82)

*DOT-direct observed therapy, HIV- human immunodeficiency virus, IQR-interquartile range, CTC-care and treatment clinic, TB-tuberculosis.*

### TB mortality rate

The total follow-up time for 10,491 children and adolescents with TB was 61,835 person-months. A total of 177 (1.69%) children and adolescents died, resulting in a crude mortality rate of (2.86, 95% CI = 2.47-3.32) per 1,000 person-months. Treatment was successful for 10,259 out of 10,491 children and adolescents (97.8%), while the remaining children and adolescents were either lost to follow-up 52 (0.5%), died 177 (1.67%), or experienced treatment failure 3 (0.03%). The lowest mortality rate per 1,000 person-months was observed in community referral settings compared to other referral types (1.22, 95% CI = 0.86-1.73). Additionally, the highest mortality rate per 1,000 person-months was seen in HIV-positive patients (11.08, 95% CI = 8.32-14.74) compared to HIV-negative patients (2.26, 95% CI = 1.90-2.68). There was no significant difference in mortality rate concerning age, sex, residence and disease classification (Table 2).

**Table 2:** Mortality rates per 1000 person-months among children and adolescents with TB (n = 10,491).

Characteristics	Person- Months	Number of Deaths	Mortality Rate Per 1000pm (95%CI)
**Overal**	**61,835**	**177**	**2.86 (2.47-3.32)**
**Age Group (Years)**			
<5	35,002	94	2.69 (2.19-3.29)
5-9	14,233	48	3.37 (2.54-4.48)
10-14	12,600	35	2.78 (1.99-3.87)
**Sex**			
Male	32,274	92	2.85 (2.32-3.50)
Female	29,561	85	2.88 (2.32-3.56)
**Residence**			
Urban	26,351	89	3.38 (2.74-4.16)
Rural	35,484	88	2.48 (2.01-3.06)
**Health facility level**			
Dispensaries	21,622	33	1.53 (1.09-2.15)
Health centres	18,193	50	2.75 (2.08-3.63)
Hospitals	22,020	94	4.27 (3.49-5.23)
**TB referral type**			
Self-referrals	22,105	58	2.62 (2.03-3.39)
CTC referrals	2,317	17	7.34(4.56-11.80)
Community referrals	25,443	31	1.22 (0.86-1.73)
Other referral types	11,970	71	5.93(4.70-7.49)
**Treatment History**			
New	61,493	176	2.86 (2.47-3.32)
Retreatment	342	1	2.92 (0.41-20.76)
**Disease classification**			
Pulmonary	45,728	138	3.02 (2.55-3.57)
Extra pulmonary	15,752	37	2.35 (1.70-3.24)
Pulmonary and extra pulmonary	355	2	5.62 (1.41-22.49)
**DOT option**			
Home based	61,246	170	2.78 (2.39-3.23)
Facility based	589	7	11.88 (5.66-24.92)
**HIV status**			
Positive	4,243	47	11.08 (8.32-14.74)
Negative	57,592	130	2.26 (1.90-2.68)

**Note:** *DOT-Direct observed therapy, HIV-Human Immunodeficiency Virus, TB- tuberculosis, CTC-Care and Treatment Centre.*

### Survival probabilities

**Fig 1:**
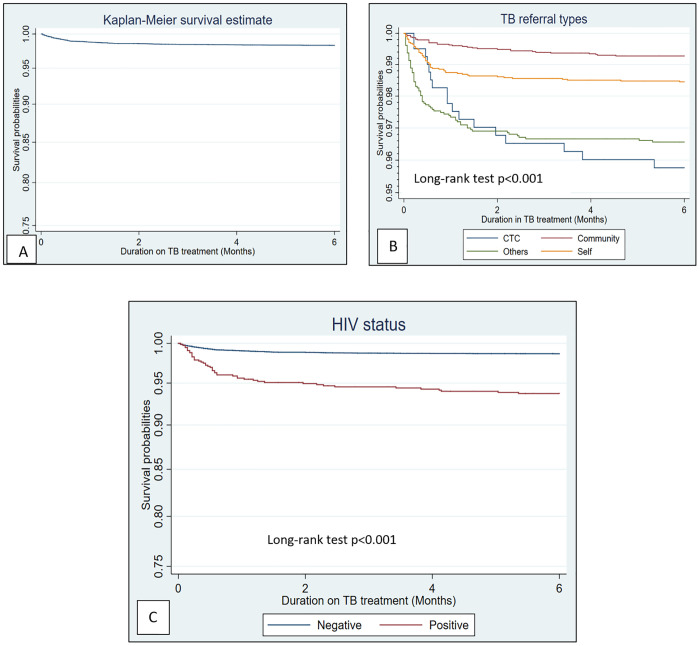
Kaplan-Meier survival curves. The figure shows the overall survival probabilities for children and adolescents with Tuberculosis (1A); the survival probabililites based on Tuberculosis referral types (1B); the survival probabilities among children and adolescents by HIV status (1C).

The overall survival probabilities among children and adolescents with TB were estimated to be 98.6%, 98.4%, and 98.3% at 2, 4 and 6 months, respectively, indicating a 0.3% relative difference between two and six months (Fig 1A). The Kaplan-Meier survival curves showed significantly lower survival probabilities for patients referred from CTC, with rates of 96.8%, 96.0% and 95.8% at 2, 4 and 6 months respectively, compared to those refered from community showed higher survival probabilities of 99.5%, 99.3% and 99.2% over the same period (Fig 1B). Patients with HIV infections had lower survival probabilities of 94.9%, 94.2% and 93.7% at 2, 4 and 6 months respectively, compared to 98.9%, 98.7% and 98.6% among HIV-negative children (p = 0.001). The relative differences survival probabilities decreased to 4.96% for TB/HIV co-infected children and adolescents compared to HIV-negative children and adolescents at the end of six months (Fig 1C).

### Factors associated with mortality

In the bivariate analysis, children and adolescents residing in urban areas had a 1.36 times higher risk of mortality (crude hazard ratio (cHR)=1.36, 95% CI = 1.01-1.82, p = 0.042) compared to children and adolescents residing in rural areas. Children and adolescents referred from CTC had nearly three times the risk (cHR = 2.74, 95% CI = 1.60-4.71, p < 0.001) compared to those who self-referred while community referrals were associated with a nearly half lower risk of mortality (cHR = 0.47, 95% CI = 0.30-0.72, p = 0.001). Children and adolescents with co-infection of TB/HIV presented an almost fivefold risk of mortality (cHR = 4.79, 95% CI = 3.43- 6.69, p < 0.001) compared to non-HIV/TB cases. Community referrals exhibited a low risk of mortality.

After adjusting for potential confounders and other variables, the independent risk factors for TB mortality included Community referrals were associated with a 54% lower risk of mortality (aHR = 0.54, 95% CI = 0.35-0.84, p = 0.006) versus self-referral and having TB/ HIV co-infection indicated five times the risk of mortality (aHR = 5.03, 95% CI = 3.40- 7.47, p < 0.001) compared to non-HIV/TB co-infection (Table 3).

**Table 3:** Cox regression analysis for risk factors associated with TB mortality among children and adolescents during TB treatment (n = 10,491).

Characteristics	Crude Hazard Ratio (95%CI)	P-value	Adjusted Hazard Ratio (95% CI)	P-value
**Age Group (Years)**				
<5	0.97 (0.65-1.42)	0.858	–	–
5-9	1.21 (0.78-1.87)	0.389	–	–
10-14	1			
**Sex**				
Male	0.99 (0.74-1.33)	0.949	–	–
Female	1			
**Residence**				
Urban	1.36 (1.01-1.82)	**0.042**	1.18 (0.87-1.59)	0.283
Rural	1		1	
**Health facility level**				
Dispensaries	1		1	
Health centres	1.79 (1.15-2.78)	**0.009**	1.51 (0.97-2.35)	0.069
Hospitals	2.78 (1.87-4.13)	**<0.001**	1.95 (1.29-2.93)	**0.001**
**TB referral type**				
Self-referrals	1		1	
CTC referrals	2.74 (1.60-4.71)	**<0.001**	0.72 (0.38-1.35)	0.302
Community referrals	0.47 (0.30-0.72)	**0.001**	0.54 (0.35-0.84)	**0.006**
Other referral types	2.25 (1.59-3.18)	**<0.001**	1.98 (1.39-2.82)	**<0.001**
**Treatment History**				
New	1		1	
Retreatment	1.03 (0.14-7.34)	0.978	0.55 (0.08-3.99)	0.555
**Disease classification**				
Pulmonary	1		1	
Extra pulmonary	0.78 (0.54-1.12)	0.177	0.82 (0.57-1.18)	0.287
Pulmonary and extra pulmonary	1.85 (0.46-7.49)	0.386	2.01 (0.50-8.17)	0.326
**DOT option**				
Home based	1		1	
Facility based	4.13 (1.94-8.81)	**<0.001**	3.32 (1.55-7.13)	**0.002**
**HIV status**				
Positive	4.79 (3.43-6.69)	**<0.001**	5.03 (3.40-7.47)	**<0.001**
Negative	1		1	

## Discussion

Our study determined the mortality rate, survival probabilities, and factors associated with mortality among children and adolescents receiving treatment for TB in Tanzania. We revealed a mortality rate of around 3 per 1,000 person-months. The mortality rate was significantly higher among children and adolescents to those with TB and HIV co-infection than their counterparts. A lower mortality rate was observed among children and adolescents referred from the community compared to other referral types. The independent risk factors for death among children and adolescents with TB included being referred from other types of referrals and having TB/ HIV co-infection. Patients referred from CTC and TB/HIV co-infection had lower survival probabilities compared to their counterparts. These findings call for the urgent need for targeted interventions to enhance TB survival, particularly for patients referred from CTC, and TB/HIV co-infected children and adolescents, to reduce mortality and improve the positive treatment outcomes.

The overall mortality rate of 2.86 per 1,000 person-months found in this study in children and adolescents under 15 years was lower than the one reported in Ethiopia [21]. We found that 1.69% of children and adolescents died, slightly higher than program data, indicating a 1.5% mortality rate in 2020 [18]. The findings were still lower than those in Botswana, Kenya, Nigeria and Ethiopia [6,17,21,22]. The low TB mortality rate could be explained by enhanced public health initiatives and awareness campaigns that have improved prevention, early detection, and treatment of TB in children [23]. A large sample size used at the national level could also contribute to the low mortality rate.

The findings of this study indicate that low survival probabilities are particularly prevalent among TB/HIV co-infected children and adolescents. Our study demonstrates a higher survival probability in TB/HIV co-infected children and adolescents compared to a study conducted in Kenya, which reported a 4.5% mortality rate and lower survival probability [17]. Improved access to and adherence to ART could enhance survival rates [24]. This can be attributed to the significantly enhanced TB/HIV integration services in the country and the improved care provided to patients at the facility level due to the decentralization of TB services [25,26]. We found that the TB treatment success rate in children and adolescents was 98%, which exceeded the WHO treatment success rate target of 91% in the 2021 cohort [27] and surpassed the results of a study conducted in Botswana [6]. This could be due to more vigorous follow-up of children undergoing treatment and improved TB programs in the country.

The study found that other referral types had nearly twice the risk of death compared to self-referral. Our findings align with studies conducted in Tanzania and Nigeria [22,28]. This may be attributed to children referred from non-program-linked clinics, traditional healers, or pharmacies who could experience delays in diagnosis due to initial misdiagnosis or a lack of adequate diagnostic facilities [15,28]. This could be with a well structured patient referral system in the community to the facility by a well trained community supporters ensuring comprehensive care and follow-up for all children with TB. Furthermore, we also found that children referred from the community had a 46% lower risk of death compared to self-referral. Several studies have indicated that community-based approaches can enhance TB care and decrease mortality rates in children [29,30]. Integrating TB care into existing child health programs to improve outcomes is essential.

In our study, similar to other studies, we found that TB/HIV co-infection had higher risk of death compared to HIV-negative children. This aligns with studies done in Botswana, Kenya, Nigeria, Ethiopia, South Africa and Tanzania [6,17,21,22,31–33]. This phenomenon can be attributed to the fact that children have underdeveloped immune systems, especially those co-infected with TB and HIV, who are more likely to develop severe forms of the disease, leading to death [21]. These findings underscore the urgent need for the immediate implementation of integrated TB/HIV services to reduce the mortality rate, as demonstrated in studies conducted in Nigeria and Malawi [22,34].

We were unable to discuss the results of higher mortality rate and strong association observed in our study between the facility vs community and those of DOTs under facility based vs community based due to strong likelihood of confounded by the unmeasured severity of the disease.

Our study has several limitations. Since we used secondary data from the national TB program database, Lack of clinical and laboratory HIV data (e.g., CD4 count, WHO/CDC stage). Absence of information on diagnostic delays. No key data on TB treatment adherence. Missing socioeconomic and parental education data. Additionally, we lacked information on the severity and other co-morbidities of TB in children and adolescents referred from primary health facilities. Lack of the final outcome (death) from children who were lost to follow-up, which could potentially lead to an underestimation of TB mortality. However, using a large sample of all children with TB enrolled in treatment from every region of mainland Tanzania renders the study representative of children receiving treatment in that region.

## Conclusion

There is a notable difference in TB mortality based on age, referral system, and HIV co-infection. Integrating TB services with child healthcare programs and strengthening differentiated service delivery models can improve survival rates. We recommend that targeted interventions in high-risk areas are crucial for reducing TB mortality.

## Supporting information

S1 File List of study participants. (XLSX)
